# Retrospective Evaluation of Subpleural Consolidations Using Lung Ultrasound in 634 Dogs and 347 Cats

**DOI:** 10.3390/ani15040549

**Published:** 2025-02-13

**Authors:** Katarzyna Kraszewska, Michał Gajewski, Søren Boysen, Natalia Buda

**Affiliations:** 1 Vetcardia Veterinary Clinic, Kijowska 11, 03-743 Warsaw, Poland; michalgajewski1980@gmail.com; 2 Faculty of Veterinary Medicine, University of Calgary, Calgary, AB T2N 1N4, Canada; srboysen@ucalgary.ca; 3 Simulation Laboratory of Endoscopic and Minimally Invasive Techniques, Medical University of Gdansk, 80-210 Gdańsk, Poland; natabud@gumed.edu.pl

**Keywords:** consolidation, color Doppler, vascularity, bronchogram

## Abstract

Background: Human literature describes vascular patterns in various types of lung consolidations; however, these changes have not been analyzed in dogs and cats. This retrospective analysis of medical records aimed to describe vascular patterns observed in the airless subpleural lung tissue of dogs and cats and to compare these patterns to various lung consolidations and clinical diagnoses according to parenchymal and vascular criteria described in human literature. Animals: This study included 347 cats and 634 dogs. Methods: This was a retrospective study encompassing medical records obtained between 2018 and 2023. Lung ultrasound was performed in dogs and cats and cases with different sonographically identified airless subpleural lung tissue were selected. Airless subpleural lung tissue was categorized into five types of consolidations: shred, nodule, wedge sign, mass, and tissue sign. Further classification was based on the parenchymal criteria, the presence or absence of bronchograms and the vascular patterns identified within these regions. Bronchograms were classified as air bronchograms (dynamic and/or static), fluid bronchograms, mixed fluid and air bronchograms, and/or absence of bronchograms. Vascular patterns were classified as tree-like, residual, chaotic, “*vascular sign*”, and whether they were continuous with and extended from the chest wall or not. Conclusions and Clinical Importance: It is possible to identify and characterize airless subpleural lung tissue in cats and dogs based on the parenchymal and vascular characteristics.

## Simple Summary

Lung ultrasound examinations were conducted on dogs and cats, selecting cases with various sonographically identified airless subpleural lung tissue. Airless subpleural lung tissue was categorized into five types of consolidations: shred, nodule, wedge sign, mass, and tissue sign. Further classification was based on parenchymal criteria, the presence or absence of bronchograms, and the vascular patterns observed within these regions.

## 1. Introduction

The exponential use of ultrasound, the availability of devices, and the popularization of point-of-care ultrasonography (POCUS) make lung ultrasound (LUS) examination a rapidly evolving diagnostic modality [1,2]. In veterinary medicine, there is limited literature describing the utility of LUS in diagnosing parenchymal lung diseases associated with airless subpleural lung tissue (ASLT) in small animal patients [3,4,5,6,7]. By contrast, LUS has been thoroughly studied in human medicine and is considered an essential tool for increasing diagnostic accuracy in dyspneic patients [8,9]. According to newly published human guidelines, the first diagnostic approach in patients with acute dyspnea should include thoracic ultrasound [10]. It allows differential diagnosis between potentially life-threatening conditions such as pulmonary edema and bronchopneumonia and can help assess the severity of lung injury [10,11,12,13]. This retrospective study analyzed ASLT using B-mode and color Doppler, according to parenchymal and vascular criteria.

The primary objective was to analyze and describe ASLT in dogs and cats according to their shape and margins, along with parenchymal and vascular criteria. The secondary objective was to determine if sonographic assessment of vascular criteria within ASLT is feasible in dogs and cats and determine if vascular criteria differ between shred, tissue, nodule, wedge sign, and mass lesions.

## 2. Materials and Methods

### 2.1. Study Population

This retrospective study included cats and dogs that presented to the Vetcardia cardiorespiratory clinic in Warsaw between May 2018 and December 2023. The patient database was mined for all patients who had both echocardiographic and non-echocardiographic thoracic ultrasound performed. A total of 45,148 patients were identified. All non-canine or feline patients and cases without ASLT in the description were excluded, leaving 2157 patients. Cases with incomplete records that did not include a final diagnosis or follow-up were also excluded, leaving 634 dogs and 347 cats.

### 2.2. Ultrasound Machine Settings and Probe Selection

All thoracic ultrasound examinations were performed using GE Vivid IQ machines; various phased-array probes (12 MHz, 6 MHz, or 3 MHz) were utilized for echocardiography, while LUS was performed with a multifrequency linear probe (12 L). Assessment of ASLT was performed using the linear probe using both “lung” and “thyroid” presets. The “lung” preset has harmonics turned off, uses the lowest frequency setting (6 MHz), has persistence set at zero, and a focal point positioned centrally within the ASLT [14,15,16]. The “thyroid” preset has harmonics and spatial compound imaging turned on, utilizes the high-frequency range of the probe, has a higher dynamic range, and has persistence set at 3; the preset was fine-tuned in each patient by positioning the focal point at the center of the ASLT. The “lung” preset creates a coarser image with better visibility of vertical artifacts, while the thyroid preset allows more detailed visualization of the echogenicity of airless lung tissue. The assessment of vascularity using Color or Power Doppler (CD/PD) mode was performed with both presets. The Doppler window was focused over regions of ASLT to detect flow signals [17]. The blood flow in a vessel was seen as a persistent area of color signal with a tubular, curvilinear, or branching distribution in real-time; color signals that persisted in the same location during the respiratory cycle were considered to be blood flow signals and not due to interference [17]. The CD/PD mode was set to the higher frequency of the Doppler mode and low pulse repetition frequency, which corresponded to a Nyquist limit of 0.07–0.09 m/s.

### 2.3. LUS Examination Technique

Six veterinarians performed echocardiography and LUS on dogs referred for cardiac evaluation. Formal echocardiography was performed in accordance with “best practice” guidelines based on ACVIM consensus, and problem-oriented echocardiography [18,19]. Lung ultrasound was performed at the discretion of the echocardiographer as a point-of-care test. Three of them had >15 years of experience in echocardiography (including KK, MG), were trained in LUS by a human specialist (NB) and had over 5 years of experience in LUS; one had >5 years of experience in echocardiography, was trained in LUS by a human specialist (NB), and had >2 years of experience in LUS, and one had >5 years’ experience in echocardiography and >2 years of experience in LUS. The animals were examined in either sternal or lateral recumbency, depending on the severity of dyspnea. A combination of alcohol and gel was used as the coupling agent. The fur was not clipped.

**Figure 1:**
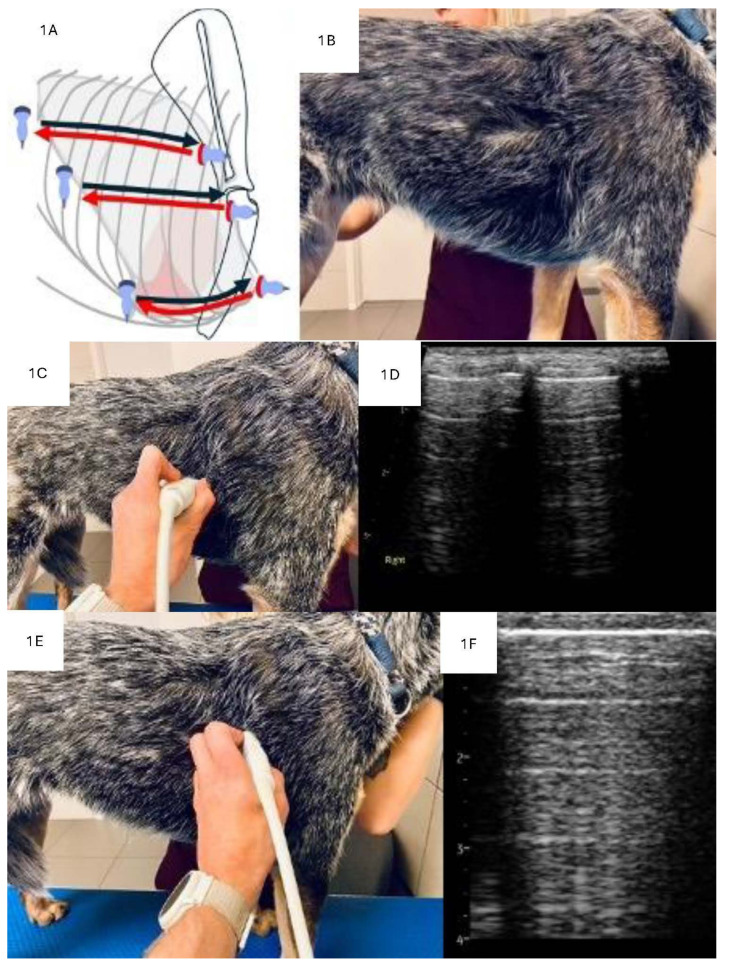
Lung ultrasound examination technique. (**A**) Schematic representation of the location of the 3 scanning lines on a dog’s thorax. Red and black arrows described the direction of the sliding of the transducer. (**B**) Photo of a dog with the fur parted and alcohol applied at the level of the middle scan line. (**C**) Position of the transducer perpendicular to the ribs (frontal plane) with corresponding sonogram (**D**). (**E**) Position of the transducer parallel to the ribs (transverse plane) with corresponding sonogram (**F**).

Lung ultrasound examination was performed with a sliding protocol involving the placement of the linear probe in three horizontal lines on each hemithorax, as previously described by Kraszewska at all (Figure 1A–C,E) [3]. Although it was not possible to determine the time to complete LUS due to the retrospective nature of the study, it typically takes 5–10 min to complete the described protocol.

### 2.4. Analysis of Consolidations in 2D

The ASLT was assessed in 2D mode according to its shape, margins, and parenchymal criteria. Whenever ASLT was described, its location on the left or right hemithorax was recorded, and for the statistical analysis, the lungs were divided into three zones: cranial, middle, and caudal (approximately 1/3 of the thoracic cavity length for each of the zones). The size of the ASLT did not influence its classification; however, it was measured and reported in two dimensions: depth (the distance between the pleural line and the lower margin of the consolidation) and width (the diameter of the consolidation at the level of the pleural line) for monitoring purposes.

Terminology from previously published human and veterinary studies was used to describe the shape of airless lung tissue, which is a broad term encompassing all non-aerated subpleural regions of the lung. ASLT was initially classified as a different type of subpleural consolidations (i.e., airless lung tissue created by pathologic processes in the lung parenchyma). The latter was further divided into the tissue (T) sign, shred (S) sign, the wedge (W) sign, and the nodule (N) sign; also, for the sake of this study, an additional category of a mass (M) was created [20,21,22,23]. The mass sign was differentiated from the nodule sign by the echogenicity of the airless lung tissue; the nodule sign was described as a round hypoechoic consolidation with smooth margins, while the mass sign was characterized as having a heterogeneous echogenicity and smooth margins. The characteristics of various types of ASLT are presented in Table 1. In some patients, a combination of different consolidation types was observed during the examination within one area of a non-aerated lung. These were classified as complex lesions, i.e., tissue and mass (T/M), shred and nodule (S/N), and other combinations [24]. Complex lesions could exist together in the lung lobe and were often classified as a secondary change in the lung as a consequence of the primary pathology (i.e., a mass as one type of consolidation and a tissue sign peripheral to the mass as a secondary lesion due to associated resorption atelectasis, as the sonomorphology of the atelectasis is similar to tissue sign but the volume of the lung tissue is reduced it was named as an atelectatic tissue-like pattern—ATLP).

Furthermore, when present, the presence of air bronchograms was noted and subclassified within regions of ASLT. In the human literature, three types of bronchograms exist: dynamic air bronchogram, static air bronchogram, and fluid bronchogram [8]. Dynamic air bronchograms are defined as hyperechoic structures visible in inspiration and disappearing on expiration; static air bronchograms represent the presence of air in the bronchial tree that is visible throughout both phases of respiration, and fluid bronchograms are formed due to the presence of fluid in the bronchial tree [8]. The consolidation was categorized as lacking a bronchogram when no visible hyperechoic (air) or hypoechoic (fluid) tree-like structure resembling a bronchial tree was visible within the consolidation. If more than one type of bronchogram was visible within a consolidation, these were described as complex findings for statistical analysis, e.g., dynamic and static air bronchogram, fluid bronchogram and the absence of bronchogram, and other combinations.

### 2.5. Analysis of Consolidations in Color/Power Doppler Mode

**Figure 2:**
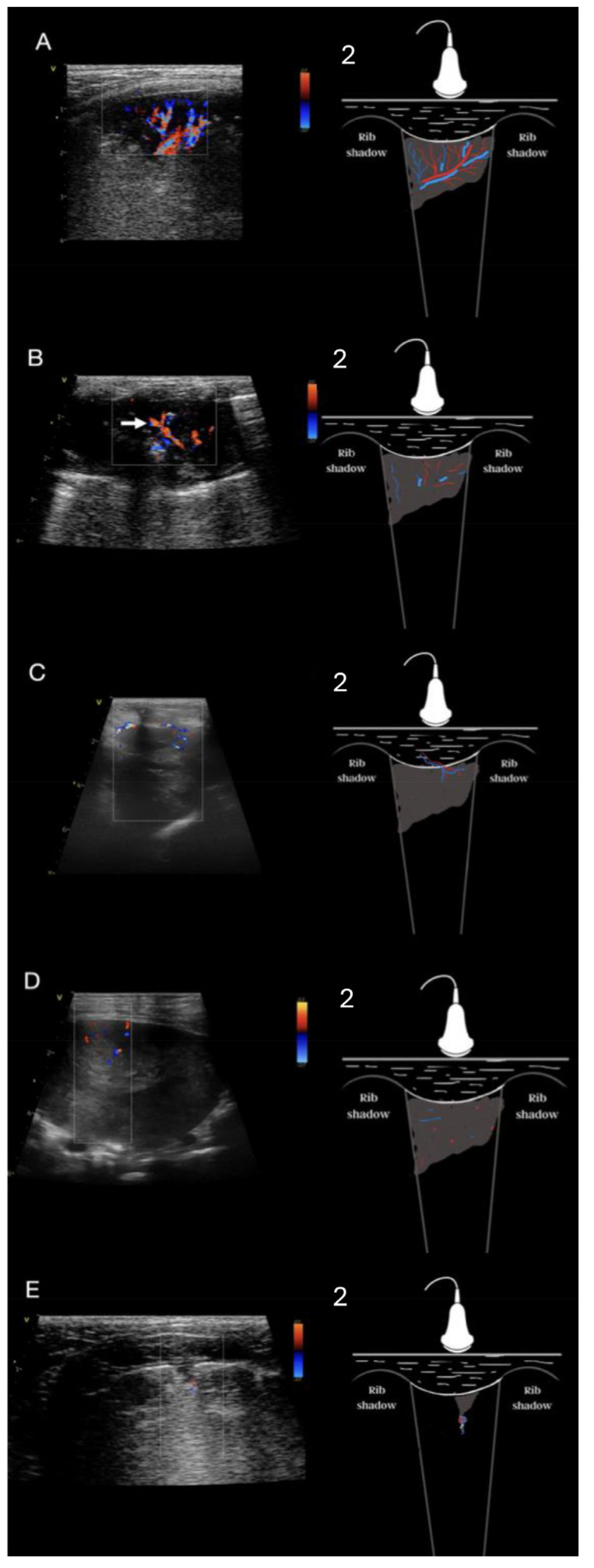
(**A**). Linear probe, lung preset, longitudinal view. Still image, color Doppler imaging of the anatomical tree—like vascularization in a dog with pneumonia. Corresponding schematic diagram showing the tree-like vascularization within a region of consolidation—transverse view. (**B**). Linear probe, lung preset, longitudinal view. Still image, color Doppler imaging of the residual vascularization (white arrow) within a region of consolidation. Corresponding schematic diagram showing a residual vascularization pattern within a region of consolidation—transverse view. (**C**). Linear probe, thyroid preset, longitudinal view. Still image, color Doppler imaging of a penetrating vascular pattern extending from the chest wall in a dog with a tumor. Corresponding schematic diagram showing a penetrating vascular pattern extending from the chest wall—transverse view. (**D**). Linear probe, thyroid preset, longitudinal view. Still image, color Doppler imaging of a chaotic vascular pattern within a region of consolidation in a dog with a tumor. The diagram shows the chaotic vascularization in consolidation—transverse view. (**E**). Linear probe, lung preset, longitudinal view. Still image, color Doppler imaging of the “*vascular sign*” in consolidation. Corresponding schematic diagram showing a “*vascular sign*” within a region of consolidation—transverse view.

Regions of ASLT were also classified according to the vascular criteria using the CD/PD mode. The presence or absence of blood flow in the non-aerated lung tissue and the spatial structure of the vascularization were assessed [25,26]. Five distinct vascular patterns were described within the ASLT: anatomical tree-like vascularization, residual vascularization, chaotic vascularization, vascularization penetrating from the chest wall into the consolidation, and blood flow amputation, referred to as the “*vascular sign*”. Tree-like vascularization is characterized by prominent, dilated blood vessels branching toward the lung’s periphery, creating images that resemble a tree (Figure 2A, Video S1). Residual vascularization is described as the presence of tree-like vessels but significantly less prominent and present only in some portions of the airless lung tissue (Figure 2B, Video S2). Chaotic vascularization is created by tiny blood vessels coursing through the consolidation in various directions without any resemblance to the normal anatomy of the lung (Figure 2D). Penetrating vascularization is characterized by one or more vessels connecting the intercostal arteries with the consolidation (Figure 2C, Video S3). The “*vascular sign*” (Figure 2E, Video S4) is believed to result from vessel occlusion by embolic material [3,26,27]. Abrupt cessation of blood flow at the “tip (i.e., the narrow, centrally located end) of the consolidation can be seen in CD/PD. In some cases, the blood flow profile was assessed using pulsed wave Doppler mode [28] to differentiate residual and chaotic vascularization [29]. Chaotic vascularization is characterized in human medicine as having a low velocity and low resistance flow profile in pulsed wave Doppler analysis [17,25,29,30].

### 2.6. Diagnosis

Depending on the presumptive diagnosis, various additional tests were performed, including echocardiography, thoracic radiographs (TXR), computed tomography (CT), bronchoscopy with bronchoalveolar lavage (BAL), fine needle aspiration (FNA), and cytology, histopathology, bacterial cultures, complete blood count and serum biochemistry, C-reactive protein level, D-dimers, and autopsy. The final diagnoses included pneumonia, interstitial lung disease (ILD), pulmonary neoplasia, metastatic disease, granuloma, abscess, acute respiratory distress syndrome (ARDS), pulmonary thromboembolism (PTE), congestive heart failure (CHF), and atelectasis. CHF was diagnosed based on an increased resting respiratory rate above 30/min, tachycardia and results of a full echocardiographic examination including 2D, M-mode, Simpson method of disc, and diastolic function assessment. Metastatic and tumor diagnoses were confirmed by radiographs and/or CT, oncologist interpretation of FNA and/or histopathology examination, and subsequent response to treatment. A diagnosis of PTE was made based on D-dimer analysis, the presence of spontaneous echocontrast in the right atrium, and autopsy results in euthanized animals. Pneumonia was confirmed through CBC and/or CRP results, TXR at the referring clinic (TXR was repeated only in chronic symptoms or in cases unresponsive to treatment), BAL with bacteriology, and bronchoscopy, or a combination of the above. In a few cases, a positive reaction to antibiotic treatment was considered supportive of pneumonia [6]. Granuloma was diagnosed with FNA or when consolidations resolved during treatment based on serial LUS examination [3]. Animals diagnosed with ARDS had severe bilateral diffuse changes with spared regions on LUS, hypoxia, and no evidence of left-sided CHF based on echocardiography.

### 2.7. Statistical Analysis

Statistical analyses were performed using the IBM SPSS Statistics 29 package. Frequency analysis and basic descriptive statistics were performed. Chi-square tests of independence were used for comparisons with nominal variables and a Kruskal–Wallis test for quantitative variables. The level of significance was set at α = 0.05. Standardized residuals with values lower than −1.96 and higher than 1.96 were considered statistically significant. Based on the values of standardized residuals, the range of *p* values was determined, where the value in the ranges 1.96 < standardized residual < 2.58 stands for *p* values < 0.050; 2.58 < standardized residual < 3.09 equals *p* < 0.010, and 3.09 < standardized residual is *p* < 0.001.

## 3. Results

### 3.1. Characteristics of the Study Group

The study included 981 cases: 55% males, 45% females, 64.6% dogs and 35.4% cats. Age ranged from 1 month to 20.3 years (M = 10.63; SD = 4.44). Dog breeds varied but mixed breed (28.2%) and Yorkshire Terriers (14.7%) were over-represented. Domestic Shorthair (DSH) (38.9%) and mixed breed (26.5%) were the most common cat breed. Detailed percentage distributions of breeds are presented in Table 2.

### 3.2. Analysis of the Consolidations

The distribution and occurrence of consolidations are reported in Table 3. The most common simple consolidation was the shred sign (47.1%), while the least common was the wedge sign (2.6%); among complex consolidations, the combination of a shred and tissue sign (14.8%) were most often identified, with the combination of the nodule and wedge sign being the least commonly identified (0.1%).

### 3.3. Analysis of Bronchograms

The classifications of bronchograms in ASLT are presented in Table 4. There was a statistically significant difference between the type of bronchogram identified within different types of ASLT. The observed effect was strong (Vc > 0.50). Dynamic bronchograms were reported more frequently with shred signs than other types of airless lung tissue (*p* < 0.001). Within tissue signs, static and fluid bronchograms, and a combination of static and fluid bronchograms, were most often observed (*p* < 0.001). Within mass, nodule, and wedge signs, the absence of bronchograms was most commonly observed (*p* < 0.001). For complex lesions, a mixed type of bronchogram was observed more often within the combined shred and tissue sign. In these consolidation types, dynamic and static, or dynamic, static, and fluid bronchograms were also observed. These mixed bronchograms are frequently observed in cases of non-interlobular consolidation. Fluid bronchograms are typically located in the peripheral regions of the consolidation, while static and dynamic air bronchograms manifest in areas where the consolidation interfaces with aerated lung tissue [20]. In the combined mass and nodule complex lesions, the absence of bronchograms was most often observed, while the static and the absence of bronchograms were most often reported within the combined shred and nodule complex lesions.

### 3.4. Analysis of Vascularity

The results of vascularity for ASLT regions are presented in Table 5, with examples shown in Figure 2A–E. The analysis showed a statistically significant difference between the compared types of airless lung tissue with respect to vascularization (Vc > 0.50). Tree-like vascularization was most often observed in shred signs (*p* < 0.001). In mass consolidations, the most common type of vascularity was chaotic and penetrating from the chest wall, along with a combination of the aforementioned types (*p* < 0.001). Within nodule signs, chaotic vascularization was most often observed (*p* < 0.001). Residual vascularization was more frequent within tissue signs (*p* < 0.001). The “*vascular sign*” was observed significantly more often within wedge signs. In the complex lesion consisting of mass and tissue signs, chaotic vascularization and vascularization penetrating from the chest wall, or their combination were most often observed. In the complex lesion of a shred and wedge sign, the “*vascular sign*” was most frequently observed. In the complex lesion of mass and nodule sign, chaotic vascularization was observed most often while residual vascularization was observed most often in the complex shred and tissue sign lesions.

### 3.5. Analysis of Types of Consolidations in Terms of Diagnosis

There was a statistically significant difference between the types of consolidations and final presumed diagnosis (Table 6). Moreover, the obtained effect was strong (Vc > 0.50). There were 633 cases with only one classification of consolidation. The shred sign was observed most often in presumed cases of ARDS, parenchymal lung disease (PLD), pneumonia, pneumonia with PLD, and pneumonia with CHF. The mass type of consolidation was noted most often in tumors, tumors with concurrent atelectasis, and tumors with concurrent CHF. Nodule signs were seen significantly more often in cases that had metastases, granulomas, and tumors with concurrent granulomas [24]. Wedge signs were seen significantly more often in cases that had a final diagnosis of pulmonary thromboembolism (PTE), pneumonia with PTE, PTE with ARDS, and PTE with CHF. Finally, tissue signs were seen most often in cases with atelectasis, as the sonomorphology of atelectasis is similar to tissue sign but the volume of the lung tissue is reduced; therefore, it was named as an atelectatic tissue-like pattern (ATLP). ATLP was also seen in cases with a combination of atelectasis and CHF as a compression atelectasis secondary to the presence of the pleural effusion or for reasons other than CHF. 

In 348 cases, complex consolidations or two different airless tissue patterns were present. The finding of shred and tissue signs or shred and ATLP was often seen in cases diagnosed with combined atelectasis, CHF and PLD, cases with pneumonia and atelectasis, and cases with pneumonia, atelectasis and CHF [31]. Complex cases with findings of mass plus ATLP were seen in cases most often diagnosed with as having tumors with atelectasis (Video S5), and tumors with CHF (compression atelectasis). Shred and wedge signs were often seen together in cases with a presumed diagnosis of PTE, pneumonia with PTE, pneumonia with PTE and ARDS, and PTE with CHF. Mass combined with nodule signs were seen in cases suspected to have tumors with metastases. Complex cases with findings of shred sign and mass signs were seen most frequently in presumed cases with tumors and metastases, and pneumonia with abscesses.

## 4. Discussion

This study demonstrates that it is possible to describe the vascular patterns of ASLT in cats and dogs. Furthermore, classification of vascular patterns in songraphically identifiable shred, tissue, nodule, and wedge signs, and mass lesions appears to differ between different lung pathologies. This is important, as it may further assist in narrow differential diagnosis and direct further diagnostic findings. For example, in human medicine, the identification of even a single peripheral wedge sign with a “*vascular sign*” is an indicator for further diagnostic tests to confirm PTE [8]. Therefore, if present, the identification of airless lung tissue and its classification, including vascular and bronchogram patterns, should prompt consideration of further diagnostic testing in cats and dogs. However, it is important to interpret LUS findings considering all additional clinical and diagnostic findings [8,32].

ASLT may develop due to various etiologies and their vascular morphological and hemodynamic changes have been investigated in human medicine. Pulmonary vascularization in animals and humans has organ-specific characteristics due to the dual arterial supply of the lungs [25,33]. Pulmonary arteries show a tree-like pattern. Their branches move centrifugally and react to hypoxia by vasoconstriction induced by the Euler–Liljestrand reflex [25], which can explain residual blood flow in atelectasis [33,34]. The vascularity of a consolidated lung area is not possible to assess using TXR, and CT is still an expensive and often less accessible method compared to standard ultrasonography, leaving the latter as a feasible diagnostic modality. From the clinical point of view, information about vascularity, type of bronchogram, or its absence in ASLT may have clinical significance and may influence further diagnostic decisions.

In the human literature, there are four typical vascular patterns described within tissue-like lesions, which were all present in cats and dogs of the current study [25,35,36]. Anatomical tree-like vascularization is seen in humans with pneumonia, which was also the case in our study, where cats and dogs with presumed pneumonia also had anatomical tree-like patterns. The shape of those consolidations was irregular. Color Doppler blood flow tended to appear and disappear with the heartbeat in these cases. The second type of vascularity, termed residual vascularization, was found in cases of atelectasis (i.e., the tissue sign) in the current study, which is similarly reported in humans. Vascularization may be reduced by a mass compressing the vessels centrally within the region of atelectasis, vasoconstriction in cases of chronic pneumonia, or secondary to significant pleural effusion in cases with severe congestive heart failure. In tumors, residual vascularization or chaotic vascularization was present, but there was no air or fluid bronchograms noted in these cases. Neoplastic lesions often obtain nutrients from oxygen-rich blood [33,34]. The anatomical structure of tumors was destroyed in some cases, likely as a result of calcifications, which appeared as small hyperechoic round dots. The third vascularization type was identified within masses, which demonstrated vessels that traverse the thoracic wall to enter the region of lung consolidation. It is believed that neovascularization of lung masses in these cases arises from the intercostal arteries, which are rich in oxygen [37]. The last observed pattern was the “*vascular sign*”. The “*vascular sign*” is believed to result from vessel occlusion by embolic material. Abrupt cessation of blood flow at the “tip” of the consolidation can be seen on color Doppler images. The consolidation is usually triangular or basket-shaped and hypoechogenic; in the literature, it is called a “wedge sign.” The “*vascular sign*” was present in dogs infested with *Angiostrongylus vasorum* [3] and cats with various types of cardiomyopathy, causing spontaneous echocontrast in the right atrium. In one case, thromboembolic lung disease was confirmed on postmortem examination. The “*vascular sign*” was also seen in seven dogs that presented with severe dyspnea that did not have a final diagnosis, all of which were Yorkshire Terriers, which were all being treated for Cushing’s disease. These dogs were euthanized due to lack of response to treatment.

Interestingly, in this retrospective analysis, various complex lesions were described, which indicates the commonality of simultaneous comorbidities—for example, finding CHF with shred signs suggests coexisting pulmonary edema and pneumonia. Humans with CHF classically have an absence of pleural line and subplueral abnormalities [8,32]. However, we observed changes in the pleural line, most commonly in gravity-dependent lung regions, in cases with severe pulmonary edema. Despite this, the shred sign was observed in cases of CHF with coexisting pneumonia. The complex lesions consisting of mass and atelectasis were better visualized on the LUS than TRX. The identification of bronchograms and vascular patterns increased the suspicion of a neoplastic process in one case diagnosed with atelectatic lung. In this case, resorption atelectasis was identified peripheral to the neoplastic lesion.

This study has several limitations. A general drawback of color Doppler sonography for LUS is that motion artifacts secondary to respirations may interfere with the quality of color Doppler sonography. Good ultrasound settings and Doppler velocity scale adjustments are essential to visualize small vessels within lung consolidations. Doppler analysis can be challenging to perform for less experienced operators. Nevertheless, regions of airless subpleural lung tissue should be evaluated with respect to their bronchograms and vascularity whenever possible. If the patient is in severe respiratory distress or panting, LUS can be performed following patients’ stabilization and/or sedation/anxiolytics are administered. LUS should be performed as soon as possible following sedation to avoid possible atelectasis, which may occur secondary to prolonged lateral recumbency.

Another limitation of this study is that lesions that fail to reach the lung periphery will not be detected. This is because lung pathology located below the lung surface will be obscured by overlying air, with only reverberation artifacts being seen [38]. Therefore, LUS cannot rule out lung pathology that is located within deeper lung tissues. Despite this, a lot of lung pathology cases, such as metastases, will occur close to the lung surface, and will therefore be seen on LUS [39,40].

## 5. Conclusions

This study demonstrates that the analysis of the shape and characterization of bronchogram, and vascularity of ASLT is possible in cats and dogs and that findings varied between patients with different lung pathologies. This may prove helpful in differentiating underlying etiologies and directing further diagnostics, although further prospective clinical research would be required to confirm the sensitivity and specificity of these findings with relation to different disease processes. Characterization of ASLT in cats and dogs may also suggest respiratory pathology is multifactorial in origin when identified on LUS.

## Figures and Tables

**Table 1:** Characterization of different types of airless lung tissue.

Type of Airless Lung Tissue	Morphology	Types of Bronchogram	Echogenicity	Margins
Nodule	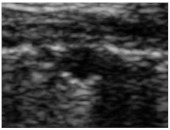	- No bronchogram	Hypoechoic	Smooth
Mass	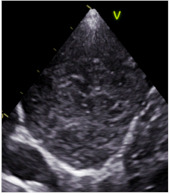	- No bronchogram	Varied	Smooth
Shred	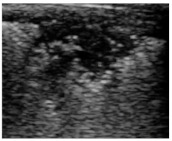	- Air dynamic - Air dynamic and static	Hypoechoic	Irregular
Tissue	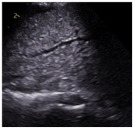	- Air static - Fluid - Air static and fluid	Varied	Smooth
Wedge	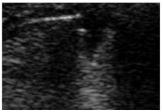	- No bronchogram	Hypoechoic	Smooth

**Table 2:** Percentage distribution of dog and cat breeds.

Species	Breed	n	%
Canine	Mix breed	179	28.20%
	Yorkshire Terrier	93	14.70%
	Cavalier King Charles Spaniel	35	5.50%
	West Highland White Terrier	29	4.60%
	Miniature Schnauzer	23	3.60%
	French Bulldog	21	3.30%
	Chihuahua	20	3.20%
	Shih Tzu	19	3.00%
	Dachshund	18	2.80%
	Jack Russell Terrier	17	2.70%
	Border Collie	15	2.40%
	Maltese	15	2.40%
	Beagle	11	1.70%
	German Shepard	11	1.70%
	Labrador Retriever	10	1.60%
	Pug	10	1.60%
	Maltipoo	8	1.30%
	Miniature Pincher	8	1.30%
	Other breeds	92	14.5%
Feline	DSH	135	38.90%
	Mix breed	92	26.50%
	Maine Coon	31	8.90%
	BSH	24	6.90%
	Persian	20	5.80%
	Syberian	14	4.00%
	Oriental	5	1.40%
	Ragdoll	5	1.40%
	NFC	5	1.40%
	Sphinx	5	1.40%
	RBC	4	1.20%
	Other breeds	9	2.10%

Abbreviations: BSH—British shorthair; DSH—domestic shorthair; n—number of observations; NFC—Norwegian forest cat; *p*—statistical significance; other breeds—breeds with six or fewer individuals; RBC—Russian blue cat.

**Table 3:** Percentage of individual types of airless lung tissue.

Consolidation Type	n	%
Shred	460	47.10%
Shred/Tissue	145	14.80%
Mass	99	10.10%
Nodule	82	8.40%
Tissue	77	7.90%
Mass/Tissue	32	3.30%
Wedge	25	2.60%
Shred/Wedge	19	1.90%
Mass/Nodule	18	1.80%
Shred/Nodule	13	1.30%
Nodule/Tissue	4	0.30%
Tissue/Wedge	4	0.30%
Nodule/Wedge	2	0.10%
All	981	100.0%

**Table 4:** Bronchograms identified within different types of airless lung tissue.

		Consolidation Type												
Bronchogram Classification		S	S/T	M	N	T	M/T	W	S/W	M/N	S/N	χ^2^	*p*	Vc
Absent (n = 219)	n	3	1	89	76	0	11	17	4	15	3	2073.85	<0.001	0.57
	%	1.0%	0.7%	93.7%	97.4%	0.0%	34.4%	94.4%	30.8%	93.8%	27.3%			
Dynamic (n = 230)	n	229	1	0	0	0	0	0	0	0	0			
	%	73.2%	0.7%	0.0%	0.0%	0.0%	0.0%	0.0%	0.0%	0.0%	0.0%			
Static (n = 42)	n	18	5	0	0	17	0	0	1	0	1			
	%	5.8%	3.5%	0.0%	0.0%	23.9%	0.0%	0.0%	7.7%	0.0%	9.1%			
Fluid (n = 16)	n	1	0	0	0	13	2	0	0	0	0			
	%	0.3%	0.0%	0.0%	0.0%	18.3%	6.3%	0.0%	0.0%	0.0%	0.0%			
Dynamic and static (n = 137)	n	59	73	1	0	1	1	0	0	0	2			
	%	18.8%	50.7%	1.1%	0.0%	1.4%	3.1%	0.0%	0.0%	0.0%	18.2%			
Static and fluid (n = 52)	n	3	11	0	0	38	0	0	0	0	0			
	%	1.0%	7.6%	0.0%	0.0%	53.5%	0.0%	0.0%	0.0%	0.0%	0.0%			
Dynamic, static and fluid (n = 54)	n	0	53	0	0	0	0	0	0	0	1			
	%	0.0%	36.8%	0.0%	0.0%	0.0%	0.0%	0.0%	0.0%	0.0%	9.1%			
Dynamic and absent (n = 11)	n	0	0	0	1	0	1	1	5	0	3			
	%	0.0%	0.0%	0.0%	1.3%	0.0%	3.1%	5.6%	38.5%	0.0%	27.3%			
Static and absent (n = 30)	n	0	0	5	1	2	17	0	3	1	1			
	%	0.0%	0.0%	5.3%	1.3%	2.8%	53.1%	0.0%	23.1%	6.3%	9.1%			

Abbreviations: M—mass; N—nodule; n—number of animals; *p*—statistical significance; S—shred; T—tissue; W—wedge; Vc—effect size; χ^2^—chi square test result.

**Table 5:** Comparison of airless lung tissue types in terms of vascularity.

		Consolidation Type												
Vascularisation Type		S	S/T	M	N	T	M/T	W	S/W	M/N	S/N	χ^2^	*p*	Vc
Tree (n = 173)	n	146	24	0	0	1	0	0	0	0	2	1011.32	<0.001	0.69
	%	88.0%	30.0%	0.0%	0.0%	2.7%	0.0%	0.0%	0.0%	0.0%	33.3%			
Residual (n = 119)	n	19	56	0	1	35	3	0	0	1	4			
	%	11.4%	70.0%	0.0%	14.3%	94.6%	13.0%	0.0%	0.0%	9.1%	66.7%			
Chaotic (n = 91)	n	0	0	59	6	0	16	0	0	10	0			
	%	0.0%	0.0%	86.8%	85.7%	0.0%	69.6%	0.0%	0.0%	90.9%	0.0%			
Chaotic and penetrating (n = 8)	n	0	0	6	0	0	2	0	0	0	0			
	%	0.0%	0.0%	8.8%	0.0%	0.0%	8.7%	0.0%	0.0%	0.0%	0.0%			
Penetrating (n = 5)	n	0	0	3	0	0	2	0	0	0	0			
	%	0.0%	0.0%	4.4%	0.0%	0.0%	8.7%	0.0%	0.0%	0.0%	0.0%			
“*Vascular sign*” (n = 31)	n	1	0	0	0	1	0	17	12	0	0			
	%	0.6%	0.0%	0.0%	0.0%	2.7%	0.0%	100.0%	100.0%	0.0%	0.0%			

Abbreviations: M—mass; n—number of observations; N—nodule; *p*—statistical significance; S—shred; T—tissue; W—wedge; Vc—effect size; vs.—“*vascular sign*”; χ^2^—chi square test result.

**Table 6:** Comparison of airless lung tissue types in terms of diagnosis.

		Consolidation Type												
Diagnosis		S	S/T	M	N	T or ATLP	M/T or ATLP	W	S/W	M/N	S/N	χ^2^	*p*	Vc
ARDS (n = 32)	n	29	0	0	0	2	0	1	0	0	0	5040.92	<0.001	0.76
	%	6.3%	0.0%	0.0%	0.0%	2.6%	0.0%	4.0%	0.0%	0.0%	0.0%			
PLD (n = 36)	n	33	2	0	0	0	0	1	0	0	0			
	%	7.2%	1.4%	0.0%	0.0%	0.0%	0.0%	4.0%	0.0%	0.0%	0.0%			
Tumor (n = 94)	n	1	0	80	5	2	4	0	0	1	1			
	%	0.2%	0.0%	81.6%	6.1%	2.6%	12.9%	0.0%	0.0%	5.6%	7.7%			
Tumor/Atelectasis (n = 42)	n	0	1	11	1	3	25	0	0	1	0			
	%	0.0%	0.7%	11.2%	1.2%	3.9%	80.6%	0.0%	0.0%	5.6%	0.0%			
Tumor/Metastasis (n = 24)	n	0	0	3	5	0	0	0	0	16	2			
	%	0.0%	0.0%	3.1%	6.1%	0.0%	0.0%	0.0%	0.0%	88.9%	15.4%			
Tumor/Granuloma (n = 1)	n	0	0	0	1	0	0	0	0	0	0			
	%	0.0%	0.0%	0.0%	1.2%	0.0%	0.0%	0.0%	0.0%	0.0%	0.0%			
Tumor/CHF (n = 3)	n	0	0	2	0	0	1	0	0	0	0			
	%	0.0%	0.0%	2.0%	0.0%	0.0%	3.2%	0.0%	0.0%	0.0%	0.0%			
Atelectasis (n = 41)	n	1	8	0	0	30	0	0	0	0	1			
	%	0.2%	5.5%	0.0%	0.0%	39.0%	0.0%	0.0%	0.0%	0.0%	7.7%			
Atelectasis/HF (n = 24)	n	0	1	0	0	23	0	0	0	0	0			
	%	0.0%	0.7%	0.0%	0.0%	29.9%	0.0%	0.0%	0.0%	0.0%	0.0%			
Atelectasis/CHF/Parenchymal lung disease (n = 1)	n	0	1	0	0	0	0	0	0	0	0			
	%	0.0%	0.7%	0.0%	0.0%	0.0%	0.0%	0.0%	0.0%	0.0%	0.0%			
Pulmonary edema (n = 1)	n	1	0	0	0	0	0	0	0	0	0			
	%	0.2%	0.0%	0.0%	0.0%	0.0%	0.0%	0.0%	0.0%	0.0%	0.0%			
Metastases (n = 65)	n	0	0	0	64	0	0	0	0	0	1			
	%	0.0%	0.0%	0.0%	78.0%	0.0%	0.0%	0.0%	0.0%	0.0%	7.7%			
Metastases/Atelectasis (n = 5)	n	0	0	0	2	0	0	0	0	0	3			
	%	0.0%	0.0%	0.0%	2.4%	0.0%	0.0%	0.0%	0.0%	0.0%	23.1%			
Pneumonia/ARDS (n = 15)	n	10	3	0	0	0	0	1	1	0	0			
	%	2.2%	2.1%	0.0%	0.0%	0.0%	0.0%	4.0%	5.3%	0.0%	0.0%			
Pneumonia (n = 281)	n	248	30	0	0	2	0	0	0	0	1			
	%	53.9%	20.7%	0.0%	0.0%	2.6%	0.0%	0.0%	0.0%	0.0%	7.7%			
Pneumonia/PLD (n = 23)	n	19	4	0	0	0	0	0	0	0	0			
	%	4.1%	2.8%	0.0%	0.0%	0.0%	0.0%	0.0%	0.0%	0.0%	0.0%			
Pneumonia/Atelectasis (n = 111)	n	17	84	1	0	6	1	0	0	0	2			
	%	3.7%	57.9%	1.0%	0.0%	7.8%	3.2%	0.0%	0.0%	0.0%	15.4%			
Pneumonia/Atelectasis/CHF (n = 2)	n	0	8	0	0	2	0	0	0	0	0			
	%	0.0%	5.5%	0.0%	0.0%	2.6%	0.0%	0.0%	0.0%	0.0%	0.0%			
Pneumonia/Abscess (n = 3)	n	0	0	1	0	1	0	0	0	0	1			
	%	0.0%	0.0%	1.0%	0.0%	1.3%	0.0%	0.0%	0.0%	0.0%	7.7%			
Pneumonia/PTE (n = 15)	n	0	0	0	0	0	0	3	12	0	0			
	%	0.0%	0.0%	0.0%	0.0%	0.0%	0.0%	12.0%	63.2%	0.0%	0.0%			
Pneumonia/PTE/ARDS (n = 2)	n	0	0	0	0	0	0	0	2	0	0			
	%	0.0%	0.0%	0.0%	0.0%	0.0%	0.0%	0.0%	10.5%	0.0%	0.0%			
Pneumonia/Granuloma (n = 1)	n	0	0	0	0	0	0	0	0	0	1			
	%	0.0%	0.0%	0.0%	0.0%	0.0%	0.0%	0.0%	0.0%	0.0%	7.7%			
Pneumonia/CHF (n = 103)	n	98	3	0	0	2	0	0	0	0	0			
	%	21.3%	2.1%	0.0%	0.0%	2.6%	0.0%	0.0%	0.0%	0.0%	0.0%			
Pneumonia/CHF/tracheal collapse (n = 1)	n	1	0	0	0	0	0	0	0	0	0			
	%	0.2%	0.0%	0.0%	0.0%	0.0%	0.0%	0.0%	0.0%	0.0%	0.0%			
PTE (n = 18)	n	1	0	0	0	0	0	14	3	0	0			
	%	0.2%	0.0%	0.0%	0.0%	0.0%	0.0%	56.0%	15.8%	0.0%	0.0%			
Metastases/ARDS (n = 5)	n	0	0	0	0	1	0	4	0	0	0			
	%	0.0%	0.0%	0.0%	0.0%	1.3%	0.0%	16.0%	0.0%	0.0%	0.0%			
PTE/Metastasis (n = 0)	n	0	0	0	0	0	0	0	0	0	0			
	%	0.0%	0.0%	0.0%	0.0%	0.0%	0.0%	0.0%	0.0%	0.0%	0.0%			
PTE/CHF (n = 2)	n	0	0	0	0	0	0	1	1	0	0			
	%	0.0%	0.0%	0.0%	0.0%	0.0%	0.0%	4.0%	5.3%	0.0%	0.0%			
Granuloma (n = 4)	n	0	0	0	4	0	0	0	0	0	0			
	%	0.0%	0.0%	0.0%	4.9%	0.0%	0.0%	0.0%	0.0%	0.0%	0.0%			
CHF (n = 4)	n	1	0	0	0	3	0	0	0	0	0			
	%	0.2%	0.0%	0.0%	0.0%	3.9%	0.0%	0.0%	0.0%	0.0%	0.0%			

Abbreviations: ATLP—atelectic tissue like-pattern; CHF—congestive heart failure; M—mass; N—nodule; *p*—statistical significance; PLD—parenchymal lung disease; PTE—pulmonary thromboembolic disease; S—shred; T—tissue; W—wedge; Vc—effect size; χ^2^—chi-square test result.

## Data Availability

The original contributions presented in this study are included in the article/Supplementary Material. Further inquiries can be directed to the corresponding author. Acknowledgments: We would like to extend our gratitude to the team at Vetcardia Clinic: Rafał Niziołek, Martyna Ochocka, Aleksandra Kolibczyńska, Anna Świerk, Kinga Erhard and Wiktoria Staszczuk.

## Supplementary Materials

The following supporting information can be downloaded at https://www.mdpi.com/article/10.3390/ani15040549/s1, Video S1. Linear probe, lung preset. Color Doppler imaging of an anatomical tree-like vascular pattern in a dog with pneumonia. Video S2. Linear probe, lung preset. Color Doppler imaging of a residual vascular pattern within a region of lung consolidation. Video S3. Linear probe, thyroid preset. Color Doppler imaging of a penetrating vascular penetrating extending from the chest wall. Video S4. Linear probe, lung preset. Color Doppler imaging showing abrupt cessation of blood flow at the “tip” of a region of a consolidation “*vascular sign*” and C-line hypoechoic triangular shape consolidation. Video S5. The linear probe with a thyroid preset exemplifies a complex form of consolidation. Power Doppler imaging reveals the blood vessels surrounding the tumor, indicating the presence of resorptive atelectasis behind the mass.

## Abbreviations

ARDS	Acute respiratory distress syndrome
ASLT	Airless subpleural lung tissue
ATLP	Atelectatic tissue-like pattern
BAL	Bronchoalveolar lavage
CD	Color Doppler
CHF	Congestive heart failure
CT	Computed tomography
FNA	Fine needle aspiration
LUS	Lung ultrasound
M	Mass
N	Nodule-type consolidation
PD	Power Doppler
PLD	Parenchymal lung disease
PTE	Pulmonary thromboembolism
TRX	Thoracic radiograph
S	Shred-type consolidation
T	Tissue-type consolidation
W	Wedge-type consolidation
